# Navigating Therapeutic Landscapes in Urothelial Cancer: From Chemotherapy to Precision Immuno-Oncology

**DOI:** 10.3390/cancers17203367

**Published:** 2025-10-18

**Authors:** Takatoshi Somoto, Takanobu Utsumi, Rino Ikeda, Naoki Ishitsuka, Takahide Noro, Yuta Suzuki, Shota Iijima, Yuka Sugizaki, Ryo Oka, Takumi Endo, Naoto Kamiya, Hiroyoshi Suzuki

**Affiliations:** Department of Urology, Toho University Sakura Medical Center, Sakura 285-8741, Japan; takatoshi.soumoto@med.toho-u.ac.jp (T.S.); rino.ikeda@med.toho-u.ac.jp (R.I.); naoki.ishitsuka@med.toho-u.ac.jp (N.I.); takahide.noro@med.toho-u.ac.jp (T.N.); yuta.suzuki@med.toho-u.ac.jp (Y.S.); shouta.iijima@med.toho-u.ac.jp (S.I.); yuuka.kizuki@med.toho-u.ac.jp (Y.S.); ryou.oka@med.toho-u.ac.jp (R.O.); takumi.endou@med.toho-u.ac.jp (T.E.); naoto.kamiya@med.toho-u.ac.jp (N.K.); hiroyoshi.suzuki@med.toho-u.ac.jp (H.S.)

**Keywords:** antibody–drug conjugates, bladder cancer, immune checkpoint inhibitors, molecular subtype, urothelial carcinoma

## Abstract

Background/Objectives: The therapeutic landscape of advanced or metastatic urothelial carcinoma (UC) has shifted from platinum chemotherapy to precision immuno-oncology. Immune checkpoint inhibitors (ICIs)—pembrolizumab, nivolumab, and avelumab—show efficacy across platinum-refractory, maintenance, and adjuvant settings, yet benefit is limited to subsets, underscoring the need for biomarkers. Antibody–drug conjugates (ADCs), notably enfortumab vedotin(EV), and targeted agents such as FGFR inhibitors further expand options. This review synthesizes current evidence and emerging paradigms to guide combinations and sequencing. Methods: We performed a narrative synthesis of peer-reviewed trials (emphasizing pivotal phase III studies), key translational investigations, and contemporary guidelines on ICIs, ADCs, HER2-directed therapies, FGFR inhibitors, molecular subtyping, and genomic profiling in UC, integrating efficacy signals, biomarker associations, and practical implications for sequencing. Results: ICIs now occupy multiple settings, but heterogeneous benefit highlights the importance of molecularly informed selection. EV alone and with pembrolizumab has produced unprecedented first-line activity, prompting a strategic shift. Molecular subtyping and genomic profiling delineate phenotypes with variable immune responsiveness and targetable vulnerabilities, enabling rational combinations and refined sequencing. Ongoing trials are evaluating next-generation ADCs, HER2-directed approaches, and dual checkpoint blockade to achieve durable, personalized disease control. Conclusions: Management of locally advanced or metastatic UC is converging on precision immuno-oncology, wherein biomarker-driven selection, molecular subtyping, and thoughtful sequencing of ICIs, ADCs, and targeted agents are central to optimizing outcomes. Active trials and translational advances are expected to refine personalized strategies and embed molecular guidance into routine care.

## Simple Summary

Urothelial carcinoma (UC) is biologically heterogeneous, and recent advances in immune checkpoint inhibitors (ICIs), antibody–drug conjugates (ADCs), and molecularly targeted therapies have materially expanded treatment options. Nevertheless, their therapeutic benefit remains confined to a subset of patients, underscoring the imperative for precision-based strategies. This review provides a biomarker-driven, narrative overview of the evolving treatment landscape in advanced UC. We highlight landmark clinical trials that have established the roles of ICIs—such as pembrolizumab, avelumab, and nivolumab—and examine the emergence of synergistic ADC-ICI combinations, notably enfortumab vedotin plus pembrolizumab. The clinical relevance of FGFR3-directed therapy and the utility of molecular subtyping in informing therapeutic decision-making are also explored. We advocate for an integrative framework that incorporates genomic profiling, immune phenotyping, and rational treatment sequencing to achieve sustained clinical benefits. These perspectives are intended to help readers navigate the transition toward biomarker-informed, personalized therapy in metastatic UC.

## 1. Introduction

Bladder cancer constitutes a considerable global health challenge, ranking as the 10th most prevalent malignancy worldwide, with over 573,000 new cases and more than 200,000 deaths annually reported [1,2,3]. Urothelial carcinoma (UC), also known as transitional cell carcinoma, comprises approximately 90% of all bladder cancer cases in developed nations. It is marked by pronounced biological heterogeneity and diverse clinical behavior. Although the majority of UC originates in the bladder, upper tract urothelial carcinoma (UTUC)—involving the renal pelvis and ureter—accounts for 5–10% of cases and is characterized by distinct clinical and molecular features. UTUC often presents at a more advanced stage and portends a poorer prognosis than bladder cancer [2,4].

Bladder cancer is conventionally classified into non–muscle-invasive bladder cancer (NMIBC) and muscle-invasive bladder cancer (MIBC), with NMIBC representing approximately 70–75% of initial diagnoses [5,6]. NMIBC is generally associated with favorable outcomes, with 5-year survival rates approaching 90% when treated with transurethral resection of bladder tumor followed by intravesical Bacillus Calmette–Guérin immunotherapy [5,6]. However, up to 20% of NMIBC cases progress to MIBC, which carries a markedly more aggressive clinical trajectory and poorer prognosis [7]. MIBC is frequently associated with micrometastatic dissemination and disease recurrence despite definitive local therapy, with 5-year survival ranging from 38% in locally advanced cases to as low as 6% in metastatic disease [3,7]. Similarly, patients with localized UTUC typically undergo radical nephroureterectomy; however, postoperative renal function decline often precludes cisplatin-based chemotherapy, highlighting the necessity of optimized perioperative strategies [4].

**Figure 1:**
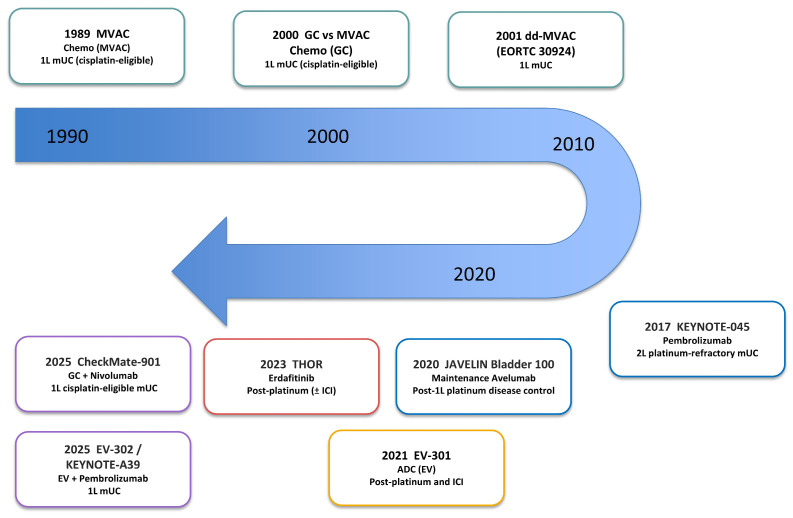
Treatment Progress in Metastatic Urothelial Carcinoma: A Chronological Timeline of Pivotal Therapies and Trials. ADCs: antibody–drug conjugates; ddMAV: dose-dense methotrexate, vinblastine, doxorubicin, and cisplatin; EV: enfortumab vedotin; GC: gemcitabine-cisplatin; ICI: immune checkpoint inhibitors; mUC: metastatic urothelial carcinoma; MVAC: methotrexate, vinblastine, doxorubicin, and cisplatin.

For decades, platinum-based chemotherapy—initially methotrexate, vinblastine, doxorubicin, and cisplatin (MVAC), later replaced by gemcitabine–cisplatin (GC)—has served as the mainstay of systemic therapy for advanced bladder cancer [8,9]. While these regimens yield clinical benefit in select patients, they are associated with considerable toxicity and limited durability responses, with nearly 50% of patients rendered ineligible for cisplatin due to age or comorbidities [3,10,11]. Over the past decade, the introduction of immune checkpoint inhibitors (ICIs), such as atezolizumab and pembrolizumab, has fundamentally reshaped therapeutic paradigms, demonstrating efficacy in metastatic, cisplatin-ineligible, and even earlier disease states [12,13,14]. Simultaneously, advances in molecular characterization have unveiled distinct tumor subtypes and actionable genetic alterations, driving the development of targeted agents (e.g., erdafitinib) and antibody–drug conjugates (ADC) (e.g., enfortumab vedotin [EV]) [15,16] (Figure 1).

The emergence of precision immuno-oncology—combining biomarker-informed patient selection with immune modulation—offers substantial promise for enhancing treatment efficacy and personalizing therapy. Nevertheless, several obstacles persist, including therapeutic resistance, the absence of robust predictive biomarkers, and the need for optimized sequencing and combination strategies.

This review provides a comprehensive synthesis of the evolving therapeutic landscape in UC, encompassing both bladder and upper tract disease. We examine the historical framework of cytotoxic chemotherapy, delineate the current immunotherapy standards, and explore emerging precision-based approaches. Through this perspective, we advocate for a paradigm shift toward personalized, biomarker-driven management of urothelial carcinoma.

## 2. Role of Chemotherapy

The systemic management of advanced and metastatic bladder cancer has historically relied on platinum-based cytotoxic chemotherapy, with MVAC emerging as the pioneering regimen in the 1980s [8]. Early clinical studies demonstrated that MVAC conferred superior efficacy over cisplatin monotherapy and other combinations, achieving a median overall survival (OS) of approximately 12.5 months alongside elevated objective response rates (ORR) [8]. As a result, MVAC was established as the gold standard for decades, both in the neoadjuvant and metastatic settings [3,10].

However, the clinical utility of MVAC was constrained by its substantial toxicity profile. High-grade hematologic toxicities were frequently observed, with neutropenic fever and mucositis contributing to a treatment-related mortality rate of up to 4% in early trials [8]. To address these limitations while preserving therapeutic efficacy, GC was introduced and directly compared to MVAC in a pivotal multinational phase III trial by von der Maase et al. [9]. This study revealed comparable OS (13.8 vs. 14.8 months), time to progression, and ORR (49% vs. 46%) between the regimens, while demonstrating significantly reduced rates of severe toxicities, including mucositis (1% vs. 22%) and neutropenic sepsis (1% vs. 12%) [9].

The GC regimen was rapidly adopted as a new standard of care due to its more favorable risk-benefit profile. Notably, a greater proportion of patients receiving GC completed all six planned cycles compared to those on MVAC, reflecting superior tolerability and treatment adherence [9]. Nonetheless, hematologic toxicities, particularly thrombocytopenia and anemia, remained prevalent across both regimens, emphasizing the need for further therapeutic refinement. In UTUC, systemic treatment strategies have largely paralleled those used for bladder cancer, although distinct perioperative considerations apply [4]. Since radical nephroureterectomy often results in renal function decline, neoadjuvant cisplatin-based chemotherapy is preferred when feasible. Furthermore, the POUT trial established adjuvant platinum-based chemotherapy as a standard approach that significantly improves disease-free and overall survival in this population [17].

Concurrently, dose-dense MVAC (dd-MVAC), supported by granulocyte colony-stimulating factor, was developed to enhance dose intensity while mitigating myelosuppressive effects [18]. In the EORTC 30924 trial, dd-MVAC yielded higher complete response rates and improved progression-free survival compared to conventional MVAC, with a more favorable hematologic toxicity profile [19]. Beyond the metastatic setting, MVAC has shown efficacy as neoadjuvant therapy. In the landmark SWOG-8710 trial, patients with MIBC who received three cycles of neoadjuvant MVAC followed by radical cystectomy experienced significantly prolonged median survival (77 vs. 46 months) and a higher pathologic complete response rate (pT0, 38% vs. 15%) compared to cystectomy alone [20]. More recently, the VESPER trial suggested that dd-MVAC may offer superior local control and 3-year progression-free survival relative to GC in the neoadjuvant context, particularly among medically fit patients [21]. These findings have reinforced the inclusion of neoadjuvant chemotherapy in standard guidelines for MIBC, although its adoption in real-world practice remains suboptimal due to concerns regarding patient eligibility, toxicity, and potential surgical delays.

Despite these advances, the limitations of platinum-based chemotherapy persist. Approximately half of patients with advanced urothelial carcinoma are ineligible for cisplatin owing to renal insufficiency, poor performance status, or comorbidities [3,10]. Moreover, treatment responses are often transient, with the majority of patients ultimately experiencing disease progression. Durable remissions remain rare, underscoring the imperative for novel therapeutic strategies.

In summary, the historical development of chemotherapy in bladder cancer, initiated with MVAC and refined through GC, has laid a foundational framework for systemic therapy. These regimens—while clinically effective—exemplify the enduring challenge of balancing efficacy and toxicity, thereby catalyzing the emergence of immunotherapeutic and precision-oncology approaches aimed at improving both clinical outcomes and tolerability.

## 3. Immune Checkpoint Inhibitors

The therapeutic landscape of advanced UC has been revolutionized by the advent of ICIs, particularly agents targeting the PD-1/PD-L1 axis [22,23]. Building upon the historical foundation of platinum-based chemotherapy, ICIs have introduced new avenues for extending survival, minimizing toxicity, and expanding treatment options across first-line, maintenance, and platinum-ineligible settings [14,24,25].

### 3.1. KEYNOTE-045 and the Emergence of Second-Line Immunotherapy

The phase III KEYNOTE-045 trial was a pivotal milestone, establishing pembrolizumab as a superior second-line option compared to chemotherapy in patients with platinum-refractory metastatic UC [12]. Pembrolizumab significantly improved OS (median 10.3 vs. 7.4 months; hazard ratio [HR], 0.73; *p* = 0.002), with a favorable safety profile and fewer grade ≥ 3 adverse events [12]. Importantly, clinical benefit was observed irrespective of PD-L1 expression, inaugurating a biomarker-agnostic treatment era.

### 3.2. JAVELIN Bladder 100 and the Shift Toward Maintenance Therapy

One of the most practice-changing developments in recent years emerged from the JAVELIN Bladder 100 trial, which evaluated avelumab as switch-maintenance therapy in patients achieving disease control following first-line platinum-based chemotherapy [13]. Avelumab significantly prolonged median OS (21.4 vs. 14.3 months; HR, 0.69; *p* = 0.001) in the overall population, with enhanced benefit observed in PD-L1–positive subgroups [13]. These findings firmly established avelumab maintenance therapy as the standard of care for patients attaining at least stable disease post-induction, a position now reflected in major clinical guidelines.

### 3.3. First-Line Treatment and Cisplatin Ineligibility

Cisplatin remains the preferred first-line cytotoxic agent for eligible patients. However, approximately 50% of patients are ineligible due to renal impairment, diminished performance status, or significant comorbidities [14,24,25]. For this subset, ICIs have provided an important therapeutic alternative. The FDA granted initial approvals for atezolizumab and pembrolizumab monotherapy in cisplatin-ineligible patients with high PD-L1 expression, based on promising single-arm trial data [26]. However, subsequent randomized phase III trials—IMvigor130 and KEYNOTE-361—did not demonstrate superiority of ICI monotherapy over chemotherapy in unselected populations, reinforcing the need for PD-L1 biomarker-guided patient selection [27,28].

### 3.4. Adjuvant Setting

In the postoperative context, the phase III CheckMate 274 trial demonstrated that one year of adjuvant nivolumab significantly improved disease-free survival (DFS) compared to placebo (median 20.8 vs. 10.8 months), particularly in PD-L1–positive patients, thereby establishing a new standard of care following radical surgery [29]. Extended follow-up has confirmed the durability of this benefit, and the AMBASSADOR trial recently validated DFS improvement with adjuvant pembrolizumab, independent of PD-L1 status [30]. By contrast, the IMvigor010 trial evaluating adjuvant atezolizumab failed to show DFS benefit over observation, indicating that not all PD-1/PD-L1 inhibitors confer equivalent efficacy in this setting [31]. In UTUC, the POUT trial provided level I evidence supporting adjuvant cisplatin-based chemotherapy as the standard of care, with significant gains in both DFS and OS. Conversely, subgroup analyses from ICI trials have not demonstrated a consistent benefit in UTUC, underscoring a tailored approach wherein adjuvant immunotherapy is favored for MIBC, while platinum chemotherapy remains the mainstay for high-risk UTUC [17].

### 3.5. Challenges in Biomarker Utilization

Despite the clinical success of ICIs, predictive biomarker development remains an ongoing challenge. While PD-L1 expression, tumor mutational burden (TMB), and circulating tumor DNA (ctDNA) have demonstrated potential, none possess sufficient predictive accuracy or standardization to warrant universal adoption across indications [22,23]. Consequently, most guideline recommendations continue to endorse a largely biomarker-agnostic framework for ICI use, except in the context of first-line monotherapy for platinum-ineligible patients, where PD-L1 testing retains clinical utility [14,25,32].

A nuanced appraisal of adjuvant trials: In IMvigor010 (open-label; anti-PD-L1 atezolizumab), the ITT population did not demonstrate a DFS benefit versus observation; however, ctDNA-positive patients derived DFS and OS advantages, and on-treatment ctDNA clearance was associated with improved outcomes—supporting a biology-anchored patient-selection paradigm [33]. By contrast, CheckMate 274 (double-blind; anti-PD-1 nivolumab) reported a DFS benefit in both the ITT and PD-L1–high cohorts; differences in blinding, PD-L1 assay/scoring (e.g., tumor-cell vs. immune-cell algorithms), population composition, and statistical hierarchy may underlie the discrepant readouts. In practice, clinicians should interpret adjuvant ICI evidence in the context of assay characteristics and molecular residual disease, while acknowledging that OS readouts and harmonized biomarker frameworks remain in evolution [29].

## 4. Emerging Approaches—Precision Immuno-Oncology

The therapeutic paradigm of UC is undergoing rapid transformation through the integration of molecularly targeted therapies and immuno-oncology within personalized treatment frameworks. While ICIs have revolutionized disease management, their efficacy is confined to a subset of patients. Precision immuno-oncology aims to transcend these limitations by leveraging biomarker-guided patient selection, molecular subtyping, and rational combinatorial approaches [34].

### 4.1. FGFR3 Alterations and Targeted Therapy

Among the most extensively characterized genomic aberrations in UC are activating mutations and fusions involving fibroblast growth factor receptor 3 (FGFR3), occurring in approximately 15–20% of metastatic cases and up to 40% of upper tract tumors [35]. Notably, FGFR3 alterations are comparatively enriched in UTUC relative to bladder UC (around 25–40% in contemporary series), thereby reinforcing the precision-oncology rationale for FGFR inhibition—exemplified by erdafitinib—in this subset [35,36,37,38]. Erdafitinib, a pan-FGFR tyrosine kinase inhibitor, received FDA approval based on the phase II BLC2001 trial and was subsequently validated in the phase III THOR study [16]. In Cohort 1 of the THOR trial, patients with FGFR3/2-altered metastatic UC who had progressed on prior platinum-based chemotherapy and ICI therapy were randomized to receive erdafitinib or investigator’s choice chemotherapy [16]. Erdafitinib significantly improved overall survival (12.1 vs. 7.8 months; HR, 0.64; *p* = 0.005) and progression-free survival (5.6 vs. 2.7 months; HR, 0.58; *p* < 0.001), with an enhanced objective response rate of 45.6% versus 11.5% [16].

Importantly, FGFR3 alterations are predominantly associated with the luminal papillary (LumP) molecular subtype, characterized by low immune cell infiltration and diminished PD-L1 expression—features that may contribute to resistance to ICIs [39,40]. This association underscores the therapeutic relevance of FGFR inhibition in ICI-refractory settings. The THOR trial reinforces the clinical utility of comprehensive molecular profiling and supports routine FGFR testing in metastatic disease [39].

### 4.2. Molecular Subtypes and Immunotherapy Response

Comprehensive transcriptomic analyses have established a consensus classification of MIBC into six molecular subtypes: LumP, luminal non-specified (LumNS), luminal unstable (LumU), stroma-rich, basal/squamous (Ba/Sq), and neuroendocrine-like (NE-like) [39,41,42]. These biologically and clinically distinct entities exhibit unique genomic alterations, immune microenvironments, and therapeutic susceptibilities (Table 1).

LumP tumors are characterized by frequent FGFR3 mutations or fusions and KDM6A alterations, indicative of papillary pathway activation. They typically exhibit low PD-L1 expression, sparse CD8+ T-cell infiltration, and an “immune-cold” phenotype, resulting in limited responsiveness to ICIs. However, they demonstrate marked sensitivity to FGFR inhibition; erdafitinib has shown significant clinical benefit in this subset [39,42,43]. Emerging preclinical and translational evidence suggests that FGFR blockade may reverse immune exclusion and restore sensitivity to PD-1 inhibition [40].

LumNS tumors constitute a smaller subset, exhibiting luminal differentiation with increased stromal and immune infiltration, including fibroblasts, B cells, and T cells [39]. This subtype is enriched for PPARG amplifications and ELF3 mutations. Although specific therapeutic trials are lacking, their immune-infiltrated phenotype implies partial ICI responsiveness, potentially constrained by stromal-mediated immunosuppression [39,42,43]. The hybrid biological features of LumNS tumors warrant combinatorial strategies targeting both tumor-intrinsic pathways and the tumor microenvironment.

LumU tumors display marked genomic instability, harboring frequent TP53 and ERCC2 mutations alongside ERBB2 amplifications. These tumors are defined by high cell-cycle activity, an APOBEC-driven mutational profile, and the highest TMB among luminal subtypes [39,41]. Such features are associated with enhanced sensitivity to cisplatin-based chemotherapy and increased likelihood of response to ICIs [39,42,43]. Retrospective analyses from ICI trials indicate that LumU tumors frequently achieve higher response rates, supporting a combined chemo-immunotherapy approach in this subgroup.

Ba/Sq tumors are clinically aggressive and commonly present with advanced T stage and squamous differentiation. They express basal keratins (KRT5/6, KRT14), CD44, and transcriptional regulators such as ΔNp63, while lacking luminal markers [39,43,44]. These tumors exhibit high EGFR expression, robust antigen presentation machinery, and elevated levels of immune checkpoints including PD-L1 and CTLA-4 [39,42,43]. The T-cell–inflamed microenvironment, with prominent CD8+ T cell and NK cell infiltration, confers strong responsiveness to cisplatin-based neoadjuvant chemotherapy and ICIs. Their EGFR dependence suggests potential benefit from EGFR-targeted therapy.

Stroma-rich tumors are defined by abundant fibroblasts and myofibroblasts and exhibit a TGF-β-driven immune-excluded phenotype [39,42,43]. Despite heavy infiltration by immune and stromal cells, CD8+ T cells remain confined to the peritumoral stroma, limiting their access to malignant cells and blunting ICI efficacy. These tumors are associated with primary resistance to PD-1/PD-L1 blockade. Combinatorial regimens incorporating TGF-β inhibition with ICIs are currently under clinical investigation [45].

NE-like tumors are distinguished by biallelic inactivation of TP53 and RB1 and overexpression of neuroendocrine markers such as chromogranin and synaptophysin. These tumors share molecular and clinical features with small-cell carcinoma and are rare but highly aggressive, with poor prognosis [39,41]. Platinum–etoposide chemotherapy remains the mainstay of treatment, paralleling protocols for small-cell lung cancer. Although PD-L1 expression is generally low, retrospective studies suggest that a subset of NE-like tumors may respond to ICIs, potentially owing to high proliferative activity and elevated mutational burden [42].

### 4.3. Integrative Precision Immunotherapy Strategies

Beyond PD-L1 expression, novel biomarkers including TMB, gene expression signatures, and ctDNA are under active investigation [22,23]. TMB-high tumors, frequently observed within LumU and Ba/Sq subtypes, have shown enhanced responses to ICIs across multiple studies [39,42]. ctDNA analysis enables non-invasive detection of minimal residual disease and evolving resistance mechanisms, offering promise for both surveillance and personalized treatment stratification.

The convergence of molecular subtyping, immune phenotyping, and genomic profiling is fostering rational, integrative treatment strategies in UC. Promising directions include the following: targeted therapy plus ICIs, combining FGFR inhibitors with ICIs in FGFR3-altered tumors that have relapsed following checkpoint blockade [40]; subtype-directed therapies, customizing treatment based on molecular subtype, for instance, utilizing EGFR inhibitors in Ba/Sq tumors and HER2-targeted agents in luminal subtypes (with Ba/Sq often overexpressing EGFR, and luminal tumors more frequently harboring HER2 alterations) [46]; stroma-rich tumors, which are marked by high angiogenic signaling and may benefit from anti-angiogenic interventions [45]; and transcriptional and epigenetic reprogramming, employing epigenetic modulators and other agents to convert immunologically "cold" tumors into "hot," immune-infiltrated phenotypes [47]. For example, oncolytic viruses and epigenetic therapies can reshape the tumor microenvironment to enhance T-cell recruitment and immune activation [48].

Primary and acquired resistance to ICIs arises from both tumor-intrinsic and microenvironmental mechanisms. (i) TGF-β-driven immune exclusion typifies stroma-rich tumors, wherein CD8+ T cells are physically sequestered within peritumoral stroma and fail to engage cancer cells, resulting in primary resistance to PD-1/PD-L1 blockade [49,50,51]. (ii) Defects in the IFN-γ pathway (e.g., JAK1/2 loss-of-function) [52] and (iii) disruption of antigen presentation (e.g., B2M inactivation or MHC class I down-regulation) underlie patterns of acquired resistance that frequently emerge under immune pressure [53]. These mechanisms align with molecular phenotypes: LumP (immune-cold) favors primary non-response; stroma-rich exhibits TGF-β—mediated exclusion [54]; and Basal/Squamous (inflamed) often responds to ICIs yet may subsequently develop adaptive resistance [55]. For ADCs, resistance may reflect target down-regulation or heterogeneity (e.g., dynamic NECTIN-4 expression), payload resistance/efflux (e.g., Monomethyl auristatin E (MMAE) handling and drug-export pumps), altered intracellular trafficking, or lineage plasticity; the relative contribution of these processes may vary by subtype context and prior ICI exposure [55]. Collectively, this framework rationalizes combination strategies (e.g., anti-TGF-β plus ICI) and sequencing that anticipates resistance biology.

To synthesize current efforts and limitations, Table 2 summarizes proposed biomarkers-ctDNA (MRD), PD-L1 assays, TMB/APOBEC, molecular-subtype signatures, NECTIN-4, and FGFR3-linking each to associated therapies, predictive signals, and practical constraints (assay variability, timing, generalizability). Exploratory ctDNA data suggest enrichment of benefit with adjuvant atezolizumab in ctDNA-positive MIUC, whereas TMB/APOBEC conveys a population-level (non-deterministic) signal for ICI benefit [33].

## 5. Combination Strategies and Sequential Therapy

Recent progress in the therapeutic management of advanced UC has emphasized the optimization of antitumor efficacy through rational combination regimens and strategic treatment sequencing. Recognizing the limitations of monotherapy—whether chemotherapy or ICIs, numerous clinical trials have explored synergistic combinations aimed at enhancing response rates, extending survival, and improving tolerability. Of particular note are combinations involving chemotherapy with ICIs and ADCs with ICIs, both of which have emerged as promising treatment paradigms.

### 5.1. Chemotherapy Plus Immune Checkpoint Inhibitors

The scientific rationale for combining chemotherapy with ICIs lies in the potential of chemotherapy to induce immunogenic cell death, thereby priming the tumor microenvironment for enhanced immune activation [56].

CheckMate-901 (nivolumab + cisplatin/gemcitabine vs. cisplatin/gemcitabine alone in cisplatin-eligible patients) was the first phase III study to demonstrate statistically significant improvements in both PFS and OS with the addition of an ICI to first-line chemotherapy. At a median follow-up of approximately 34 months, the combination arm achieved a median OS of 21.7 months versus 18.9 months for chemotherapy alone (HR 0.78; *p* = 0.02), with a corresponding improvement in PFS (HR 0.72; *p* = 0.001), establishing nivolumab plus gemcitabine–cisplatin as a new standard first-line treatment for advanced UC [57].

Two additional pivotal phase III trials—IMvigor130 and KEYNOTE-361—assessed the addition of atezolizumab and pembrolizumab, respectively, to first-line platinum-based chemotherapy [58]. IMvigor130 (atezolizumab + platinum/gemcitabine vs. chemotherapy alone) showed modest PFS improvement but failed to achieve statistical significance for OS at interim analysis [27]. KEYNOTE-361 (pembrolizumab + chemotherapy vs. chemotherapy alone) likewise did not meet predefined OS superiority thresholds [28].

These trials underscored the mechanistic rationale for chemo-immunotherapy combinations while highlighting the challenges in translating incremental clinical benefits into statistically significant outcomes in biomarker-unselected populations.

### 5.2. Enfortumab Vedotin Plus Pembrolizumab: A Synergistic ADC-ICI Approach

The strongest evidence for combination therapy in UC comes from EV—a Nectin-4–directed ADC—combined with pembrolizumab. In the phase Ib/II EV-103 trial of cisplatin-ineligible, treatment-naïve patients with locally advanced or metastatic UC, the confirmed ORR was 73.3%, including 15.6% complete responses [59]. Median DOR and OS were 25.6 and 26.1 months, respectively [59].

This synergy likely reflects EV-induced immunogenic cell death that augments antigen presentation and T-cell priming, further potentiated by PD-1 blockade [60]. Clinical responses occurred across PD-L1 subgroups and in patients with liver metastases, supporting broad applicability [58].

Accordingly, the phase III EV-302 (KEYNOTE-A39; NCT04223856) directly compared EV plus pembrolizumab with platinum–gemcitabine as first-line therapy in cisplatin-eligible and -ineligible patients [61,62]. In this randomized, global trial (*n* = 886), EV plus pembrolizumab improved all primary endpoints: median OS 31.5 vs. 16.1 months (HR 0.47; *p* < 0.0001) and median PFS 12.5 vs. 6.3 months (HR 0.45; *p* < 0.0001). The confirmed ORR was 67.7% vs. 44.4%, with complete responses 29% vs. 12% [61,62]. Benefits were consistent across subgroups irrespective of cisplatin eligibility, PD-L1 status, or visceral involvement. Safety was manageable and consistent with known toxicities of each agent, with fewer hematologic events than chemotherapy but higher rates of cutaneous toxicity, peripheral neuropathy, and hyperglycemia. Conceptually, EV represents a field-defining ADC exemplar, highlighting the importance of target-expression dynamics (NECTIN-4) and payload selection (MMAE) for next-generation ADC design and ICI sequencing.

### 5.3. Sequencing Strategies: Chemotherapy → ICI → ADC

**Figure 2:**
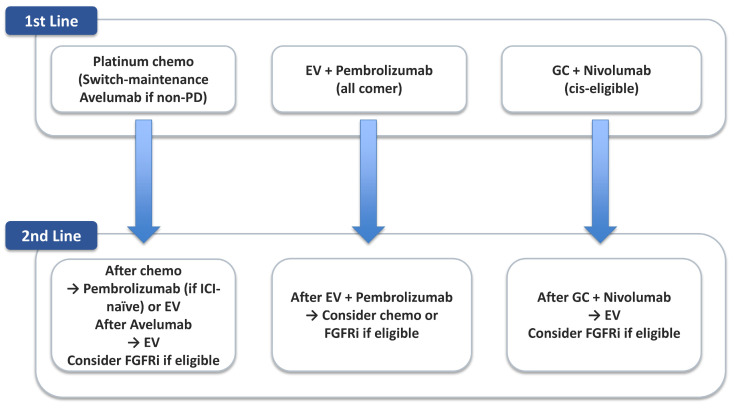
Contemporary Treatment Sequencing in Metastatic Urothelial Carcinoma. chemo: chemotherapy, cis: cisplatin, EV: enfortumab vedotin, FGFRi: fibroblast growth factor receptor inhibitor, GC: gemcitabine–cisplatin, ICI: immune checkpoint inhibitors, PD: progressive disease.

To better illustrate current treatment sequencing, Table 3 summarizes the principal therapeutic options available in the first-line setting for advanced UC. These include cisplatin-based combination chemotherapy for eligible patients, carboplatin-based regimens for those ineligible for cisplatin, maintenance immunotherapy following chemotherapy, and, most recently, ADC plus ICI combinations such as EV with pembrolizumab. Figure 2 shows a decision map that links these choices to downstream sequencing.

Sequential treatment approaches are also gaining prominence. The JAVELIN Bladder 100 trial established avelumab maintenance therapy following first-line platinum-based chemotherapy as a new standard of care, providing an overall survival benefit for patients who achieved disease control [13]. This underscores the importance of optimizing the timing of ICI introduction—whether concurrently with chemotherapy or in the post-chemotherapy maintenance phase.

Furthermore, in patients who progress after chemotherapy and ICIs, ADCs such as EV (demonstrated in EV-301) remain effective. In EV-301, enfortumab vedotin significantly improved OS (12.88 vs. 8.97 months; HR, 0.70; *p* = 0.001) and PFS (5.55 vs. 3.71 months; HR, 0.62; *p* < 0.001) compared to chemotherapy [15]. Collectively, these data establish EV as a standard of care after platinum and PD-1/PD-L1 therapy, cementing ADC monotherapy as a validated pillar in sequencing strategies for advanced UC.

## 6. Clinical Trials and Future Directions

The future landscape of metastatic UC is being actively reshaped by an expanding pipeline of clinical trials evaluating novel immunotherapy combinations, ADCs, and targeted therapies, both in biomarker-defined cohorts and broader patient populations (Table 4).

Among the most notable is SGNDV-001 (NCT05911295), a global phase III trial assessing the HER2-targeted ADC disitamab vedotin in combination with pembrolizumab as first-line therapy for patients with HER2-expressing advanced UC. If successful, this study could establish a new standard for the approximately 30% of UC patients with HER2 overexpression or amplification [63], representing a shift toward biomarker-guided initial therapy. This approach is supported by the known prevalence of HER2 in UC and the precedent set by EV plus pembrolizumab.

In SWOG S1937 (NCT04579224), a phase III study, the combination of eribulin and gemcitabine is being evaluated as second-line therapy in patients who have progressed following platinum chemotherapy and PD-1/PD-L1 inhibition. This trial addresses an important therapeutic gap in a heavily pretreated population, comparing the novel microtubule inhibitor eribulin to standard second-line chemotherapy. The trial is ongoing, with approximately 55% of the planned enrollment accrued.

In the frontline setting, the NILE study (NCT03682068) is evaluating durvalumab in combination with platinum–gemcitabine, with or without the anti–CTLA-4 antibody tremelimumab, compared to standard chemotherapy. This trial may elucidate the added value of dual checkpoint blockade and define benchmarks for chemo–immunotherapy regimens in biomarker-unselected mUC populations—an area where prior studies (IMvigor130, KEYNOTE-361) produced equivocal findings.

The phase II TAS-120-203 trial (NCT04601857) is exploring futibatinib, an irreversible FGFR1–4 inhibitor, in combination with pembrolizumab. Stratifying patients by FGFR alteration status, this study examines whether targeted FGFR inhibition can enhance immunotherapeutic responses in genomically defined subsets, and potentially sensitize resistant tumors to checkpoint blockade.

Collectively, these ongoing trials reflect the biological heterogeneity of mUC and the necessity of multifaceted therapeutic strategies. They exemplify the integration of ADCs into earlier lines of therapy, the expansion of checkpoint inhibitors into combination regimens, and the tailored use of targeted agents to overcome resistance. Importantly, the global scope of these trials ensures broader applicability and accelerates the path toward regulatory approval. As results mature, they are expected to inform next-generation treatment algorithms and propel the field toward a fully personalized, biomarker-driven model of care.

Cellular immunotherapies and cancer vaccines are emerging precision modalities in mUC. Although clinical development in mUC remains nascent, multiple platforms are being evaluated in human trials with biomarker-restricted enrollment (e.g., HLA-A*02:01/MAGE-A4 for TCR-T, HER2 for dendritic-cell vaccines). Table 5 provides a concise summary of therapeutic modalities and their corresponding targets/biomarkers.

## 7. Conclusions

The therapeutic landscape of advanced UC has shifted toward biomarker-anchored strategies. EV-based combinations and chemo-IO regimens now constitute broadly applicable first-line backbones across cisplatin-eligibility strata, whereas FGFR inhibition provides precision options for FGFR2/3-altered diseases. To extend durable benefits, we advocate resistance-informed sequencing—anticipating TGF-β-driven immune exclusion, IFN-γ–pathway defects, and a loss of antigen presentation—and rational ADC development that accounts for target-expression dynamics (e.g., NECTIN-4) and payload pharmacology (e.g., MMAE). Near-term priorities include harmonization of PD-L1 assays, rigorous evaluation of ctDNA-defined MRD to guide adjuvant and surveillance decisions, and clinical maturation of cell-therapy and vaccine platforms in mUC. Ultimately, integrating genomic profiling, immune phenotyping, and pragmatic trial designs will be essential to realize durable, patient-centered outcomes.

## Figures and Tables

**Table 1:** Consensus Molecular Subtypes of Muscle-Invasive Bladder Cancer—Genomic and Immune Features with Therapeutic Implications.

Molecular Subtype	Genomic Hallmarks	Immune Phenotype	Therapeutic Implications
LumP	FGFR3 mutations or fusions KDM6A mutations	Non–T cell–inflamed PD-L1 low CD8 low	Poor response to ICIs High sensitivity to FGFR inhibitors
LumNS	PPARG amplifications ELF3 mutations enriched	Intermediate infiltration Stromal suppression	Intermediate ICI responsiveness ICI + TGF-β or stroma-targeted agents may be needed
LumU	TP53 and ERCC2 mutations ERBB2 (HER2) amplifications APOBEC/TMB-high	Inflamed luminal Highest TMB among luminal	Good response to platinum chemotherapy and ICIs
Ba/Sq	EGFR high Basal keratins (KRT5/6, KRT14) and ΔNP63 Squamous differentiation	T cell–inflamed PD-L1/CTLA-4 high	Highly sensitive to cisplatin-based chemotherapy and to ICIs EGFR-targeted therapies could benefit a subset
Stroma-rich	TGF-β/stromal programs Fibroblasts/myofibroblasts	Immune-excluded (T cells trapped in stroma)	Intrinsic resistance to ICIs ICI + TGF-β inhibitors or anti-angiogenic agents are under investigation
NE-like	TP53 and RB1 loss NE markers	Low PD-L1 and T-cell infiltration	Platinum-etoposide chemotherapy Limited ICI responses

Ba/Sq: Basal/Squamous; ICI: immune checkpoint inhibitors; LumNS: Luminal Non-specified; LumP: Luminal Papillary; LumU: Luminal Unstable; NE: neuroendocrine; TMB: tumor mutational burden.

**Table 2:** Proposed Biomarkers in Urothelial Carcinoma.

Biomarker	Typical Assay	Therapy/Decision Anchor	Predictive Signals
ctDNA	Tumor-informed plasma NGS	Adjuvant ICI selection; MRD-guided surveillance; trial enrichment	ctDNA+ after cystectomy shows benefit signal with adjuvant atezolizumab; on-treatment clearance → better outcomes
PD-L1 IHC	28-8/SP263 (tumor-cell) SP142 (immune-cell)	Eligibility/expected benefit for selected ICI regimens	Context-dependent; strongest signals in specific settings
TMB/APOBEC	Panel-based TMB (mut/Mb) APOBEC signatures	Population-level likelihood of ICI benefit	High TMB/APOBEC associates with higher response probability
NECTIN-4	IHC/RNA proxies	ADC (EV) context	Broad expression supports activity Reduced expression may track resistance
FGFR2/3 alterations	Tumor NGS (DNA/RNA)-mutations/fusions	FGFR inhibitor candidacy	Altered tumors show drug sensitivity

ADC: antibody–drug conjugate, EV: enfortumab vedotin, FFPE: formalin-fixed paraffin-embedded, MRD: molecular residual disease, ICI: immune checkpoint inhibitor, IHC: immunohistochemistry, TMB: tumor mutational burden.

**Table 3:** First-line Therapeutic Regimens in Metastatic Urothelial Carcinoma.

Regimens	Treatment Setting	Median OS (mo)	Median PFS (mo)	ORR (%)
MVAC (Methotrexate + Vinblastine + Doxorubicin + Cisplatin) [8]	First-line metastatic (cisplatin-eligible)	~12.5 mo	~8.3 mo	~46%
GC (Gemcitabine + Cisplatin) [9]	First-line metastatic (cisplatin-eligible)	13.8 mo vs. 14.8 mo (MVAC) HR 1.04, *p* = 0.89	7.7 mo vs. 8.3 mo (MVAC)	49% vs. 46% (MVAC)
Enfortumab Vedotin + Pembrolizumab [61,62]	First-line (cisplatin-eligible & -ineligible)	31.5 mo vs. 16.1 mo (chemo) HR 0.47, *p* < 0.0001	12.5 mo vs. 6.3 mo HR 0.45, *p* < 0.0001	67.7% vs. 44.4% (chemo)
Nivolumab + Gemcitabine/Cisplatin [57]	First-line (cisplatin-eligible)	21.7 mo vs. 18.9 mo (GC alone) HR 0.78, *p* = 0.02	7.9 mo vs. 7.6 mo HR 0.72, *p* = 0.001	57.6% vs. 43.1% (GC)

HR: hazard ratio; mo: months; ORRs: objective response rates; OS: overall survival; PFS: progression-free survival.

**Table 4:** Selected Ongoing or Recent Clinical Trials in Advanced Urothelial Carcinoma.

Clinical Trials	Population/Setting	Experimental Arm	Comparator Arm	Primary Endpoints
SGNDV-001 Phase III NCT05911295	HER2-positive mUC First-line therapy	Disitamab vedotin (anti-HER2 ADC) + pembrolizumab	Platinum-gemcitabine chemotherapy	OS, PFS, ORR
SWOG S1937 Phase III NCT04579224	mUC progressed after platinum and PD-1/PD-L1 inhibitor (platinum-ICI refractory)	Eribulin (novel microtubule inhibitor) + gemcitabine	Investigator’s choice chemotherapy (e.g., single-agent chemo)	OS, PFS
NILE Phase III NCT03682068	First-line metastatic mUC (all patients, biomarker-unselected)	Durvalumab (PD-L1 inhibitor) + platinum-gemcitabine ± tremelimumab (CTLA-4 inhibitor)	Platinum-gemcitabine chemotherapy	OS, PFS
TAS-120-203 Phase II NCT04601857	mUC (advanced), stratified by FGFR3 mutation status	Futibatinib (pan-FGFR1–4 inhibitor) + pembrolizumab	No direct comparator (single-arm study with FGFR-altered vs. wild-type cohort analysis)	ORR, PFS, Safety

ADCs: antibody–drug conjugates; ICIs: immune checkpoint inhibitors; mUC; metastatic urothelial carcinoma; ORRs: objective response rates; OS: overall survival; PFS: progression-free survival.

**Table 5:** Selected Cell Therapy and Vaccines for Advanced Urothelial Carcinoma.

Clinical Trials	Modality	Target/Biomarker
Phase I NCT04044859	Cell Therapy TCR-T	MAGE-A4; HLA-A*02
Phase I NCT06318871	Cell Therapy CIML NK + low-dose IL-2	Polyclonal NK (memory-like)
Phase II NCT05248789	Oncolytic Virus oHSV-2	Oncolytic HSV-2 (GM-CSF-expressing)
Phase I NCT03359239	Personalized Vacccine + Atezolizumab	Patient-specific neoantigens
Phase I NCT01730118	Dendritic Cell Vaccine targeting HER2	HER2

CIML NK: Cytokine-Induced Memory-Like Natural Killer, GM-CSF: Granulocyte-Macrophage Colony-Stimulating Factor, HLA: Human Leukocyte Antigen, MAGE: Melanoma-associated antigen, TCR-T: T-cell Receptor–Engineered T-cell, oHSV: oncolytic Herpes Simplex Virus.

## Abbreviations

The following abbreviations are used in this manuscript:

ADC	antibody–drug conjugates
Ba/Sq	basal/squamous
ctDNA	circulating tumor DNA
DFS	disease-free survival
dd-MVAC	dose-dense MVAC
EV	enfortumab vedotin
FGFR3	fibroblast growth factor receptor 3
GC	gemcitabine–cisplatin
HR	hazard ratio
LumNS	luminal non-specified
LumP	luminal papillary
LumU	luminal unstable
MIBC	muscle-invasive bladder cancer
MMAE	monomethyl auristatin E
MVAC	methotrexate, vinblastine, doxorubicin, and cisplatin
NE	neuroendocrine
NMIBC	non–muscle-invasive bladder cancer
ORRs	objective response rates
OS	overall survival
TMB	tumor mutational burden
UC	urothelial carcinoma
UTUC	upper tract urothelial carcinoma

## References

[1] Bray F., Laversanne M., Sung H., Ferlay J., Siegel R.L., Soerjomataram I., Jemal A. (2024). Global cancer statistics 2022: GLOBOCAN estimates of incidence and mortality worldwide for 36 cancers in 185 countries. CA Cancer J. Clin..

[2] Siegel R.L., Giaquinto A.N., Jemal A. (2024). Cancer statistics, 2024. CA Cancer J. Clin..

[3] Bilim V., Kuroki H., Shirono Y., Murata M., Hiruma K., Tomita Y. (2022). Advanced bladder cancer: Changing the treatment landscape. J. Pers. Med..

[4] Escobar D., Wang C., Suboc N., D’souza A., Tulpule V. (2025). Diagnosis and management of upper tract urothelial carcinoma: A review. Cancers.

[5] Gomez Caamano A., Garcia Vicente A.M., Maroto P., Rodríguez Antolín A., Sanz J., Vera González M.A., Climent M.Á. (2021). Management of localized muscle-invasive bladder cancer from a multidisciplinary perspective: Current position of the Spanish Oncology Genitourinary (SOGUG) Working Group. Curr. Oncol..

[6] Babjuk M., Burger M., Capoun O., Cohen D., Compérat E.M., Dominguez Escrig J.L., Gontero P., Liedberg F., Masson-Lecomte A., Mostafid A.H. (2022). European Association of Urology guidelines on non-muscle-invasive bladder cancer (Ta, T1, and carcinoma in situ). Eur. Urol..

[7] Patel V.G., Oh W.K., Galsky M.D. (2020). Treatment of muscle-invasive and advanced bladder cancer in 2020. CA Cancer J. Clin..

[8] Sternberg C.N., Yagoda A., Scher H.I., Watson R.C., Geller N., Herr H.W., Morse M.J., Sogani P.C., Vaughan E.D., Bander N. (1989). Methotrexate, vinblastine, doxorubicin, and cisplatin for advanced transitional cell carcinoma of the urothelium: Efficacy and patterns of response and relapse. Cancer.

[9] von der Maase H., Hansen S.W., Roberts J.T., Dogliotti L., Oliver T., Moore M.J., Bodrogi I., Albers P., Knuth A., Lippert C.M. (2000). Gemcitabine and cisplatin versus methotrexate, vinblastine, doxorubicin, and cisplatin in advanced or metastatic bladder cancer: Results of a large, randomized, multinational, multicenter, phase III study. J. Clin. Oncol..

[10] Godlewski D., Czech S., Szpara J., Bartusik-Aebisher D., Aebisher D. (2025). A narrative review of current advances and future changes regarding bladder cancer treatment. Uro.

[11] Dobruch J., Oszczudłowski M. (2021). Bladder cancer: Current challenges and future directions. Medicina.

[12] Bellmunt J., de Wit R., Vaughn D.J., Fradet Y., Lee J.-L., Fong L., Vogelzang N.J., Climent M.A., Petrylak D.P., Choueiri T.K. (2017). Pembrolizumab as second-line therapy for advanced urothelial carcinoma. N. Engl. J. Med..

[13] Powles T., Park S.H., Voog E., Caserta C., Valderrama B.P., Gurney H., Kalofonos H., Radulović S., Demey W., Ullén A. (2020). Avelumab maintenance therapy for advanced or metastatic urothelial carcinoma. N. Engl. J. Med..

[14] Witjes J.A., Bruins H.M., Carrión A., Cathomas R., Compérat E., Efstathiou J.A., Fietkau R., Gakis G., Lorch A., Martini A. (2024). European Association of Urology guidelines on muscle-invasive and metastatic bladder cancer: Summary of the 2023 guidelines. Eur. Urol..

[15] Powles T., Rosenberg J.E., Sonpavde G.P., Loriot Y., Durán I., Lee J.-L., Matsubara N., Vulsteke C., Castellano D., Wu C. (2021). Enfortumab vedotin in previously treated advanced urothelial carcinoma. N. Engl. J. Med..

[16] Loriot Y., Matsubara N., Park S.H., Huddart R.A., Burgess E.F., Houédé N., Banek S., Guadalupi V., Ku J.H., Valderrama B.P. (2023). Erdafitinib or chemotherapy in advanced or metastatic urothelial carcinoma. N. Engl. J. Med..

[17] Birtle A., Johnson M., Chester J., Jones R., Dolling D., Bryan R.T., Harris C., Winterbottom A., Blacker A., Catto J.W.F. (2020). Adjuvant chemotherapy in upper tract urothelial carcinoma (the POUT trial): A phase 3, open-label, randomised controlled trial. Lancet.

[18] Sternberg C.N., de Mulder P.H.M., van Oosterom A.T., Fossa S.D., Giannarelli D., Soedirman J.R. (1993). Escalated M-VAC chemotherapy and recombinant human GM-CSF in patients with advanced urothelial tract tumors. J. Clin. Oncol..

[19] Sternberg C.N., de Mulder P.H.M., Schornagel J.H., Théodore C., Fossa S.D., van Oosterom A.T., Witjes F., Spina M., van Groeningen C.J., de Balincourt C. (2001). Randomized phase III trial of high-dose-intensity MVAC chemotherapy and recombinant human GM-CSF versus classic MVAC in advanced urothelial tract tumors: EORTC Protocol No. 30924. J. Clin. Oncol..

[20] Grossman H.B., Natale R.B., Tangen C.M., Speights V.O., Vogelzang N.J., Trump D.L., deVere White R.W., Sarosdy M.F., Wood D.P., Raghavan D. (2003). Neoadjuvant chemotherapy plus cystectomy compared with cystectomy alone for locally advanced bladder cancer. N. Engl. J. Med..

[21] Pfister C., Gravis G., Fléchon A., Chevreau C., Mahammedi H., Laguerre B., Guillot A., Joly F., Soulié M., Allory Y. (2022). Dose-dense MVAC or gemcitabine–cisplatin as perioperative chemotherapy for nonmetastatic muscle-invasive bladder cancer: Results of the GETUG-AFU V05 VESPER trial. J. Clin. Oncol..

[22] Roviello G., Catalano M., Santi R., Palmieri V.E., Vannini G., Galli I.C., Buttitta E., Villari D., Rossi V., Nesi G. (2021). Immune checkpoint inhibitors in urothelial bladder cancer: State of the art and future perspectives. Cancers.

[23] Yajima S., Masuda H. (2025). Immune checkpoint inhibitors and antibody–drug conjugates in urothelial carcinoma: Current landscape and future directions. Cancers.

[24] Powles T., Bellmunt J., Comperat E., De Santis M., Huddart R., Loriot Y., Necchi A., Pérez-Valderrama B., Ravaud A., Shariat S.F. (2024). ESMO clinical practice guideline interim update on first-line therapy in advanced urothelial carcinoma. Ann. Oncol..

[25] Flaig T.W., Spiess P.E., Abern M., Agarwal N., Bangs R., Boorjian S.A., Buyyounouski M.K., Chan K., Chang S.S., Friedlander T. (2024). NCCN Guidelines^®^ Insights: Bladder Cancer, Version 3.2024. J. Natl. Compr. Cancer Netw..

[26] Suzman D.L., Agrawal S., Ning Y., Maher V.E., Fernandes L.L., Karuri S., Tang S., Sridhara R., Schroeder J., Goldberg K.B. (2019). FDA approval summary: Atezolizumab or pembrolizumab for the treatment of patients with advanced urothelial carcinoma ineligible for cisplatin-containing chemotherapy. Oncologist.

[27] Grande E., Arranz J.Á., De Santis M., Bamias A., Kikuchi E., Garcia del Muro X., Park S.H., De Giorgi U., Alekseev B., Mencinger M. (2024). Atezolizumab plus chemotherapy versus placebo plus chemotherapy in untreated locally advanced or metastatic urothelial carcinoma (IMvigor130): Final overall survival analysis of a randomized, controlled, phase 3 study. Lancet Oncol..

[28] Powles T., Csőszi T., Özgüroğlu M., Matsubara N., Géczi L., Cheng S.Y., Fradet Y., Oudard S., Vulsteke C., Barrera R.M. (2021). Pembrolizumab alone or combined with chemotherapy versus chemotherapy as first-line therapy for advanced urothelial carcinoma (KEYNOTE-361): A randomized, open-label, phase 3 trial. Lancet Oncol..

[29] Galsky M.D., Bajorin D.F., Witjes J.A., Gschwend J.E., Tomita Y., Nasroulah F., Li J., Collette S., Pérez-Valderrama B., Grimm M.-O. (2023). Disease-free survival analysis for patients with high-risk muscle-invasive urothelial carcinoma from the randomized CheckMate 274 trial by PD-L1 combined positive score and tumor cell score. Eur. Urol..

[30] Apolo A.B., Ballman K.V., Sonpavde G., Berg S., Kim W.Y., Parikh R., Teo M.Y., Sweis R.F., Geynisman D.M., Grivas P. (2025). Adjuvant pembrolizumab versus observation in muscle-invasive urothelial carcinoma. N. Engl. J. Med..

[31] Bellmunt J., Hussain M., Gschwend J.E., Albers P., Oudard S., Castellano D., Daneshmand S., Nishiyama H., Majchrowicz M., Degaonkar V. (2021). Adjuvant atezolizumab versus observation in muscle-invasive urothelial carcinoma (IMvigor010): A multicentre, open-label, randomized, phase 3 trial. Lancet Oncol..

[32] Horwich A., Babjuk M., Bellmunt J., Bruins H.M., De Reijke T.M., De Santis M., Gillessen S., James N., Maclennan S., Palou J. (2019). EAU–ESMO consensus statements on the management of advanced and variant bladder cancer: An international collaborative multi-stakeholder effort under the auspices of the EAU and ESMO Guidelines Committees. Ann. Oncol..

[33] Powles T., Assaf Z.J., Degaonkar V., Grivas P., Hussain M., Oudard S., Gschwend J.E., Albers P., Castellano D., Nishiyama H. (2024). Updated Overall Survival by Circulating Tumor DNA Status from IMvigor010: Adjuvant Atezolizumab versus Observation. Eur. Urol..

[34] Xiao J., Caliri A.W., Duex J.E., Theodorescu D. (2021). Targetable Pathways in Advanced Bladder Cancer: FGFR Signaling. Cancers.

[35] Mohanty S.K., Lobo A., Mishra S.K., Cheng L. (2023). Precision Medicine in Bladder Cancer: Present Challenges and Future Directions. J. Pers. Med..

[36] Shigeta K., Matsumoto K., Kitaoka S., Omura M., Umeda K., Arita Y., Mikami S., Fukumoto K., Yasumizu Y., Tanaka N. (2024). Profiling FGFR3 Expression Based on the Immune Microenvironment in Upper Tract Urothelial Carcinoma. Eur. Urol. Oncol..

[37] Robinson B.D., Vlachostergios P.J., Bhinder B., Liu W., Li K., Moss T.J., Bareja R., Park K., Tavassoli P., Cyrta J. (2019). Upper Tract Urothelial Carcinoma Has a Luminal–Papillary T-Cell–Depleted Contexture and Activated FGFR3 Signaling. Nat. Commun..

[38] Nally E., Young M., Chauhan V., Wells C., Szabados B., Powles T., Jackson-Spence F. (2024). Upper Tract Urothelial Carcinoma (UTUC): Prevalence, Impact and Management Challenge. Cancer Manag. Res..

[39] Kamoun A., de Reyniès A., Allory Y., Sjödahl G., Robertson A.G., Seiler R., Hoadley K.A., Groeneveld C.S., Al-Ahmadie H., Choi W. (2020). A Consensus Molecular Classification of Muscle-Invasive Bladder Cancer. Eur. Urol..

[40] Okato A., Utsumi T., Ranieri M., Zheng X., Zhou M., Pereira L.D., Chen T., Kita Y., Wu D., Hyun H. (2024). FGFR Inhibition Augments Anti-PD-1 Efficacy in Murine FGFR3-Mutant Bladder Cancer by Abrogating Immunosuppression. J. Clin. Investig..

[41] Choi W., Ochoa A., McConkey D.J., Aine M., Höglund M., Kim W.Y., Real F.X., Kiltie A.E., Milsom I., Dyrskjøt L. (2017). Genetic Alterations in the Molecular Subtypes of Bladder Cancer: Illustration in the Cancer Genome Atlas Dataset. Eur. Urol..

[42] Lobo A., Collins K., Kaushal S., Acosta A.M., Akgul M., Adhya A.K., Al-Ahmadie H.A., Al-Obaidy K.I., Amin A., Amin M.B. (2024). Molecular Heterogeneity among Conventional and Subtype Histology of Urothelial Carcinoma: A Review. Histopathology.

[43] Seiler R., Ashab H.A.D., Erho N., van Rhijn B.W.G., Winters B., Douglas J., Van Kessel K., Fransen van de Putte E.E., Sommerlad M., Wang N.Q. (2017). Impact of Molecular Subtypes in Muscle-Invasive Bladder Cancer on Predicting Response and Survival after Neoadjuvant Chemotherapy. Eur. Urol..

[44] Groeneveld C.S., Pfister C., Culine S., Harter V., Krucker C., Fontugne J., Dixon V., Sirab N., Bernard-Pierrot I., de Reyniès A. (2025). Basal/Squamous and Mixed Subtype Bladder Cancers Present Poor Outcomes after Neoadjuvant Chemotherapy in the VESPER Trial. Ann. Oncol..

[45] van der Heijden M.S., Powles T., Petrylak D., de Wit R., Necchi A., Sternberg C.N., Matsubara N., Nishiyama H., Castellano D., Hussain S.A. (2022). Predictive Biomarkers for Survival Benefit with Ramucirumab in Urothelial Cancer in the RANGE Trial. Nat. Commun..

[46] Helal D.S., Darwish S.A., Awad R.A., Ali D.A., El-Guindy D.M. (2023). Immunohistochemical-Based Molecular Subtypes of Muscle-Invasive Bladder Cancer: Association with HER2 and EGFR Alterations, Neoadjuvant Chemotherapy Response and Survival. Diagn. Pathol..

[47] Pang L., Zhou F., Liu Y., Ali H., Khan F., Heimberger A.B., Chen P. (2024). Epigenetic Regulation of Tumor Immunity. J. Clin. Investig..

[48] Yang L., Gu X., Yu J., Ge S., Fan X. (2021). Oncolytic Virotherapy: From Bench to Bedside. Front. Cell Dev. Biol..

[49] Barrueto L., Caminero F., Cash L., Makris C., Lamichhane P., Deshmukh R.R. (2020). Resistance to Checkpoint Inhibition in Cancer Immunotherapy. Transl. Oncol..

[50] Zhou X., Ni Y., Liang X., Lin Y., An B., He X., Zhao X. (2022). Mechanisms of Tumor Resistance to Immune Checkpoint Blockade and Combination Strategies to Overcome Resistance. Front. Immunol..

[51] Jang A., Brown J.R. (2025). Strategies to Overcome Resistance to Enfortumab Vedotin and Pembrolizumab for Urothelial Carcinoma. Explor. Target. Antitumor Ther..

[52] Shin D.S., Zaretsky J.M., Escuin-Ordinas H., Garcia-Diaz A., Hu-Lieskovan S., Kalbasi A., Grasso C.S., Hugo W., Sandoval S., Torrejon D.Y. (2017). Primary Resistance to PD-1 Blockade Mediated by JAK1/2 Mutations. Cancer Discov..

[53] Han X., Zhang J., Li W., Huang X., Wang X., Wang B., Gao L., Chen H. (2025). The Role of B2M in Cancer Immunotherapy Resistance: Function, Mechanism, and Reversal Strategies. Front. Immunol..

[54] Mariathasan S., Turley S.J., Nickles D., Castiglioni A., Yuen K., Wang Y., Kadel E.E., Koeppen H., Astarita J.L., Cubas R. (2018). TGF-β Attenuates Tumour Response to PD-L1 Blockade by Contributing to Exclusion of T Cells. Nature.

[55] Khoury R., Saleh K., Khalife N., Saleh M., Chahine C., Ibrahim R., Lecesne A. (2023). Mechanisms of Resistance to Antibody-Drug Conjugates. Int. J. Mol. Sci..

[56] Wang Y., Fletcher R., Yu J., Zhang L. (2018). Immunogenic Effects of Chemotherapy-Induced Tumor Cell Death. Genes Dis..

[57] van der Heijden M.S., Sonpavde G., Powles T., Necchi A., Burotto M., Schenker M., Sade J.P., Bamias A., Beuzeboc P., Bedke J. (2023). Nivolumab plus Gemcitabine–Cisplatin in Advanced Urothelial Carcinoma. N. Engl. J. Med..

[58] Evmorfopoulos K., Mitrakas L., Karathanasis A., Zachos I., Tzortzis V., Vlachostergios P.J. (2023). Upper Tract Urothelial Carcinoma: A Rare Malignancy with Distinct Immuno-Genomic Features in the Era of Precision-Based Therapies. Biomedicines.

[59] Hoimes C.J., Flaig T.W., Milowsky M.I., Friedlander T.W., Bilen M.A., Gupta S., Srinivas S., Merchan J.R., McKay R.R., Petrylak D.P. (2023). Enfortumab Vedotin Plus Pembrolizumab in Previously Untreated Advanced Urothelial Cancer. J. Clin. Oncol..

[60] Fahey C.C., Clark-Garvey S., Porten S., Kamat A.M., Flaig T.W., Taylor J.A., Kim W.Y., Milowsky M.I. (2025). Mechanistic Insights and Future Directions for Enfortumab Vedotin in Urothelial Carcinoma. Curr. Oncol..

[61] Gupta S., Loriot Y., van der Heijden M.S., Bedke J., Valderrama B.P., Kikuchi E., Fléchon A., Petrylak D., De Santis M., Galsky M.D. (2025). Enfortumab Vedotin plus Pembrolizumab versus Chemotherapy in Untreated Advanced Urothelial Cancer (EV-302): Patient-Reported Outcomes. Lancet Oncol..

[62] Powles T.B., van der Heijden M.S., Loriot Y., Bedke J., Valderrama B.P., Iyer G., Kikuchi E., Hoffman-Censits J., Vulsteke C., Drakaki A. (2025). Enfortumab Vedotin plus Pembrolizumab in Untreated Advanced Urothelial Carcinoma: 2.5-Year Median Follow-Up of EV-302/KEYNOTE-A39. Ann. Oncol..

[63] Powles T.B., Grande E., Alimohamed N., Oliveira N., Sridhar S.S., Drakaki A., Kanesvaran R., Loriot Y., Necchi A., Franco S. (2025). SGNDV-001: Disitamab Vedotin with Pembrolizumab in HER2-Expressing Advanced Urothelial Carcinoma. Future Oncol..

